# Impacts of Covid-19 on mental health service provision in the Western Cape, South Africa: The MASC study

**DOI:** 10.1371/journal.pone.0290712

**Published:** 2023-08-28

**Authors:** Thandi Davies, Ingrid Daniels, Marinda Roelofse, Carol Dean, John Parker, Charlotte Hanlon, Graham Thornicroft, Katherine Sorsdahl

**Affiliations:** 1 Alan J. Flisher Centre for Public Mental Health, Department of Psychiatry & Mental Health, University of Cape Town, Rondebosch, Cape Town, South Africa; 2 Cape Mental Health, Observatory, Cape Town, South Africa; 3 Western Cape Department of Health, Western Cape Province, South Africa; 4 Department of Psychiatry & Mental Health, University of Cape Town, Rondebosch, Cape Town, South Africa; 5 Centre for Global Mental Health, Health Service and Population Research Department, and WHO Collaborating Centre for Mental Health Research and Training, Institute of Psychiatry, Psychology and Neuroscience, King’s College London, London, United Kingdom; 6 Department of Psychiatry, School of Medicine, College of Health Sciences, Addis Ababa University, Addis Ababa, Ethiopia; 7 Centre for Global Mental Health and Centre for Implementation Science, Institute of Psychiatry, Psychology and Neuroscience, King’s College London, London, England; College of Medicine of the University of Lagos, NIGERIA

## Abstract

In the context of an already large treatment gap in South Africa, this study aimed to examine how Covid-19 and the related lockdown measures affected the availability, accessibility, quality, and continuity of mental health services in the Western Cape province in South Africa. A mixed-methods design was employed, using narrative surveys, quantitative surveys, and qualitative semi-structured interviews, with 17 public mental health providers, and secondary data from the District Health Information System. We analysed and combined the data using descriptive statistics, template analysis and methodological triangulation. Results showed that Covid-19 and the lockdowns had negative impacts on mental health service provision at all levels of care, such as reduced access to services, increased stigma and discrimination, disrupted medication supply, increased workload and stress for providers, and the closure of psychosocial and therapeutic services. Innovations used by providers to mitigate these impacts included telehealth, online training, peer support groups, and community outreach. The study concludes that Covid-19 and the lockdowns exposed and exacerbated the existing gaps and challenges in mental health service provision in South Africa. Key recommendations for policy formation and response to future pandemics in the public mental health sector include: classifying psychological treatments as essential services, establishing an intersectoral mental health emergency response plan, involving mental health care users in the development of pandemic responses, creating policies for managing health emergencies in psychiatric facilities, and increasing resources for the mental health sector in South Africa. These recommendations are relevant for South Africa and other LMICs in ensuring adequate mental health care during public health emergencies.

## Introduction

Similar to other low- and middle-income countries (LMICs), the prevalence of mental health conditions in South Africa is markedly high. Estimates from South Africa indicate that at least one in six adult South Africans may be living with a mental health condition in a 12-month period [1]. Further, events at global and regional levels, such as the COVID-19 pandemic [2], climate change [3], and conflict situations are expected to further contribute to an increase in mental health conditions [4, 5]. Poverty, exposure to violence, adverse childhood experiences, and substance use [1, 6–8], well known social determinants of mental health conditions, will continue to increase the burden.

Despite this high prevalence, availability of services is poor, with the treatment gap estimated at 89% [9]. There are several contributors to this high treatment gap. For example, implementation of the South African Mental Health Policy and Strategic Plan is poor [10, 11]. In addition, in 2019, only 4.6% of the total public health budget was given to mental health care, with 86% of this allocated to inpatient care, 14% attributed to outpatient care, and only 8% to mental health care at the primary level [9]. There are also only 0.31 public sector psychiatrists and 0.97 public sector psychologists per 100 000 uninsured population [9]. This has detrimental impacts on the quality of mental health services and the availability of specialists to provide adequate care.

With the outbreak of Covid-19, the South African government implemented strict lockdowns over the ensuing 24 months to mitigate the spread of the virus and reduce burden on the health system. These were considered to be some of the most stringent lockdowns globally, and included a closure of most medical services or restriction to emergency appointments and collection of medications [12] in order to enforce social distancing and make space for Covid-19 cases. Resources were focused almost entirely on biomedical elements, with little regard for the mental health impact of the virus [13]. All individuals had to remain at home, and were only allowed to leave their residence to provide essential services, acquire food, or obtain emergency medical attention [14]. Cigarette sales were banned for five months, and alcohol for five weeks. All elective medical or surgical procedures were put on hold and community-based services were shifted to screening for Covid-19.

It has been found that stringent lockdowns during Covid-19 increased distress, decreased psychological well-being, and exacerbated the social determinants of mental illnesses, such as poverty [15]. In South Africa, Covid-19 and the subsequent lockdowns had significant impacts on mental health [13, 16–18]. A national study found that 57% of participants experienced stress, 43% fear, and 32% depression in the first week of lockdown [19], and another reported that 61.2% of participants felt stressed or anxious, and 31.6% felt sad, angry and/or frustrated during the lockdown [20]. In the Western Cape, the province in which this study was based, a survey of 860 participants in the second month of lockdown found that almost half met the diagnostic criteria for depression and anxiety [18]. Those with pre-existing mental health conditions reported higher severity of their symptoms. Symptom severity was significantly associated with distress around containment and risk of infection [18]. The increased incidence of mental health conditions and high levels of distress led to increased demand for services, while these were concurrently shut down or restricted to emergency care [12].

Yet, little research has been conducted on the impacts of the pandemic on mental health service provision and the treatment gap in South Africa, or indeed on the long-term impacts of Covid-19 on mental health. The objective of this study was to explore the consequences of Covid-19 and the related lockdowns on mental health service provision and the treatment gap in community care, primary, district, and specialist services in the Western Cape province in South Africa. It further aimed to identify examples of good practice during the lockdowns, in order to make recommendations that may be applicable for other health emergencies going forward. This study was part of a larger study titled ‘Mental health care: Adverse Sequelae of Covid-19’ (MASC), which collected similar data from six other LMICs, to identify and track adverse implications of Covid-19 for global mental health services.

## Methods

### Setting

South Africa has nine provinces, which vary considerably in size. This study focused on the Western Cape Province, which has a population of 6.84 million (Stats SA, 2019). In this province, there are 0.89 public sector psychiatrists and 1.22 public sector psychologists per 100 000 uninsured population, respectively [9]. The mental health system is largely centralised, with the majority of in-patient and outpatient services located at specialist and district hospitals in urban areas [21]. In the Cape Town Metro, there are three specialist psychiatric hospitals (referred hereafter as specialist hospitals), three district tertiary hospitals, and one regional hospital, comprising 882 mental health beds. In the rest of the province, there are three rural regional hospitals and 20 district hospitals, amounting to a total of 117 mental health beds. The study was approved by the University of Cape Town Human Ethics Research Council (Ref: 552/2020). Written informed consent was obtained from all participants.

### Study design and data collection

A mixed methods design was used to obtain data from four sources, including: 1) a narrative survey; 2) a quantitative survey, 3) qualitative semi-structured interviews; and 4) District Health Information System (DHIS) data. The study questionnaires were created by international study partners specifically for the MASC international study. A brief description of the tools follows.

1. The Narrative Survey was a qualitative tool that contained open-ended questions on Covid-19 related changes at all levels of health care under five broad themes. These were: (1) Provision of, and access to, services for people with mental illness; (2) Quality of services; (3) Delivery of health care services and systems; (4) Exemplars of good practice during the Covid-19 pandemic; and (5) Changes in mental health needs for health care workers (HCWs).
2. The Quantitative survey focused on similar categories of impacts of Covid-19, and used check boxes to collect data. The survey contained sections on: (1) Disruptions to mental health services due to Covid-19 and quality of services, (2) Level of operation of service provision; (3) Reasons for service disruption, and (4) Modifications made to service delivery.
3. The Qualitative interview enabled a deeper exploration of the effects of Covid-19 and lockdown on the provision of care and related modifications that may have been made by institutions, to supplement the survey data collected. Questions were based on the themes from the narrative survey.
4. District Health Information System (DHIS) data included numbers of admissions to specialist and district hospitals per month, and out-patient visits to specialist, district, and primary mental health services (pre-Covid-19 and during Covid-19).

Last, all stakeholders were sent the initial report from the data collected so that they could provide feedback and corroborate the evidence (member checking). A virtual meeting was then held with seven available stakeholders to present points of convergence and dissonance and to clarify findings.

### Participants

Potential participants were identified through purposive and snowball sampling of public mental health service providers and non-governmental organisations (NGOs) working in the Western Cape Province. This was done by accessing known contacts in these sectors and requesting further contacts through these participants. Approximately 30 stakeholders were approached and a total of 17 consented to participate in the study, comprising public sector servants, NGO service providers, psychiatrists, psychologists, mental health nurses, counsellors and occupational therapists. Participants were requested to complete the quantitative and narrative tools and/or participate in an interview. Five participants completed the quantitative and narrative survey tools only, eight participants participated in the semi-structured interviews only, and four participated in both. DHIS data were obtained from the Western Cape Department of Health (DOH), and included all recorded admissions and out-patient visits to mental health services from 1 November 2019 to 30 June 2021.

Survey participants were requested to populate the quantitative and narrative survey tools. Semi-structured interviews were conducted online, at a time convenient to the participants. Once all data had been obtained and analysed, a preliminary report was compiled and sent to participants. A virtual group meeting was then convened to obtain feedback on the reported findings and refine coding or clarify reported data. Ten participants attended this meeting.

### Data analysis and triangulation

Data from the narrative survey and qualitative interviews were synthesized and coded using template analysis [22] in NVivo 12. A coding template was developed from a priori themes from the narrative and qualitative tools. This was then refined through analysis of the data, and sub-themes were created from participant responses. Data from the quantitative tool was collated in Microsoft Excel. DHIS data were extracted and analysed in SPSS version 27.

All data were then analysed using ‘methodological triangulation’ [23], which involved six steps. First, we sorted the themes and data from both the qualitative and quantitative sources into categories. Second, we created a matrix where we ‘convergence coded’ to identify where there was ‘agreement, silence or dissonance’ between data from the various sources and methods. All coding was done by the lead researchers separately (TD & KS). Third, we conducted a convergence assessment to provide a global assessment on the level of convergence by the two coders. Fourth, the researchers reviewed and compared initial coding to compare the assessment of convergence and dissonance, and to determine the degree of agreement among the triangulated findings. Given the high level of agreement between the coders, a third researcher was not required to mediate disagreements. Saturation of themes and data was reached after approximately two-thirds of the data had been analysed. Fifth, the preliminary findings were presented and discussed at the virtual group meeting, to clarify findings and refine the convergence coding (89% agreement was calculated). Consensus was made after discussions on points of dissonance. This assisted in increasing the validity and trustworthiness of the research findings.

## Results

Seventeen stakeholders participated in this study. No personal details were collected besides their occupations. These are listed in Table 1 below.

**Table 1:** Participant characteristics.

Occupation	Number of participants in sample
Public sector psychiatrist	Specialist: 4, District: 3
Public sector psychologist	Specialist: 3, District: 1, Primary: 1
Public sector mental health nurse	Primary: 1
Mental health and substance abuse public sector servant	1
NGO service provider	1
Registered counsellor	1
Occupational therapist	1

Several themes emerged from the triangulation process. These were: negative impacts on care users at the community level; reductions in mental health service provision at all levels of care; interruption in the provision of therapeutic services; minimal provision for care users in legislations; increased mental health needs of health care workers; and innovations that emerged as a result of Covid-19 restrictions. The majority of the data related to the first ‘strict’ lockdown (level 5), which lasted for five weeks. Participants related that during the second and third waves of Covid-19 and associated lockdowns (levels 3 and 4), limited services were made available through slightly more flexible means. Participants were identified by numbers coded from 101 up to 117. The data is presented below according to the six primary themes derived from the template analysis.

### 1) Negative impacts on care users at the community level

The closure of the economy and restricted movements in the initial ‘hard lockdown’ led to a lack of income, food insecurity and increased vulnerability to mental illnesses for care users. Strict travel restrictions [12] and lack of money for transport impacted mental health care users’ (MHCUs) ability to access both mental and physical health services, meaning that some did not visit clinics or hospitals to fetch their medication. Primary Health Centres (PHCs) themselves shut down at times due to Covid-19, forcing MHCUs to travel further to obtain medication. Other MHCUs did not fetch their medication because they were afraid of catching Covid-19 by using public transport and from visiting clinics and hospitals, and thus relapsed.

Outpatient substance abuse and opioid detoxification centres were closed to reduce face-to-face contact and increase provision of Covid-19 beds [12]. This had severe consequences for those with substance use disorders. On top of this, the cigarette ban [24] was reported to have had a major impact on patients’ mental health. In some cases, patients then “reverted to illegal behaviour or illegal outlets to obtain cigarettes” (Specialist Hospital psychiatrist 109). Others turned to Cannabis and Methamphetamine to manage their tobacco and alcohol withdrawal, and many heroin addicts relapsed (for example, in one district hospital, 50/52 patients relapsed to heroin use) (Specialist Hospital psychiatrist 109). A particularly vulnerable group during Covid-19 were homeless MHCUs (NGO service provider 104). They were not able to receive normal services as no field visits could be done by NGOs and there was reduced access to usual food and shelter for them [24].

The enforcement of social distancing and closure of therapeutic services at all levels of care during the lockdown meant that many MHCUs no longer had peer contact at rehabilitation workshops or at out-patient therapeutic groups (Specialist Hospital psychologist 111). Patients who were admitted to specialist and district psychiatric facilities were not allowed contact with their families and support networks. These all lead to increased vulnerability and feelings of isolation (Specialist Hospital psychologist 119). During lockdown, NGOs reported ill-treatment by families and stigmatisation of MHCUs, marked increases in anxiety for some MHCUs, and arguments and physical altercations with family and community members. At times this led to involuntary admissions to specialist services (NGO service provider 104).

Despite MCHUs being a vulnerable group, there were no special communications from the government to MHCUs regarding Covid-19 and/or the potential impact on mental health. Consequently, mental health-oriented NGOs played a significant role in communicating with MHCUs and supporting them during lockdown, including the provision of food and clothing parcels. Their social workers distributed infographics via WhatsApp and other social media platforms to clients on a regular basis on Covid-19 and mental health, as well as information on services for grief and loss counselling (e.g. Cape Mental Health, South African Depression and Anxiety Group (SADAG)).

The consequences of the various lockdown restrictions led to many MHCUs experiencing relapses of their illnesses, resulting in acute admission, as described by a district psychiatrist below:

*“After those deceptively quiet weeks*, *those five deceptively quiet weeks*, *the mental health storm started*, *and it felt like a storm*. *Our patients were coming in with a variety of stresses*. *All of our patients*, *every single mental healthcare user that came in was very*, *very ill*. *Very unwell*. *And it was a combination of factors*. *It was a combination of not being treated for five weeks*. *Being with unwell families for five weeks*. *The stress of Covid*. *The inability to access your usual service*. *The adolescents were also very stressed in addition to all the other stresses that were going on*. *We were seeing very sick adolescents*. *Additionally*, *we have seen very serious suicide attempts” (Specialist Hospital psychiatrist 109)*.

In addition to the impact of the lockdown on MHCUs, an increase in mental health needs were reported among the general population. One NGO that provides telephonic counselling nationally reported that the number of calls they received monthly nearly tripled during Covid-19, rising from 15 365 calls in February 2020 to 48 563 in July 2020 [25].

### 2) Reduction in mental health service provision

Changes to provision of treatment occurred at inpatient admission level as well as at out-patient services at specialist, district and primary care (PHC) levels. The key factors causing disruption in service provision are presented in Table 2 below.

**Table 2:** Key reasons for mental health service disruption and decreased quality of care.

Reasons for disruption	Specialist hospitals	District hospitals	Primary care
Decrease admissions, raise admission thresholds, early discharges	x	x	N/A
Reduce outpatient appointments, only attend to emergencies	x	x	x
Encourage patients not to come for appointments & to stay at home	x	x	
Closure of face-to-face medical appointments (Out-Patient Department ‐ OPD)	x	x	x
Closure of therapeutic services	x	x	N/A
Mental health trained staff deployed to provide COVID-19 support	x	x	x
Conversion of psychiatric wards and bed space to COVID-19 beds	x	x	N/A
Prescribe longer prescriptions	x	x	x
Send patients straight to pharmacy	x	x	x
Unavailability/stock-out of essential medicines	x	x	x

#### 2.1. Inpatient admissions

**Fig 1:**
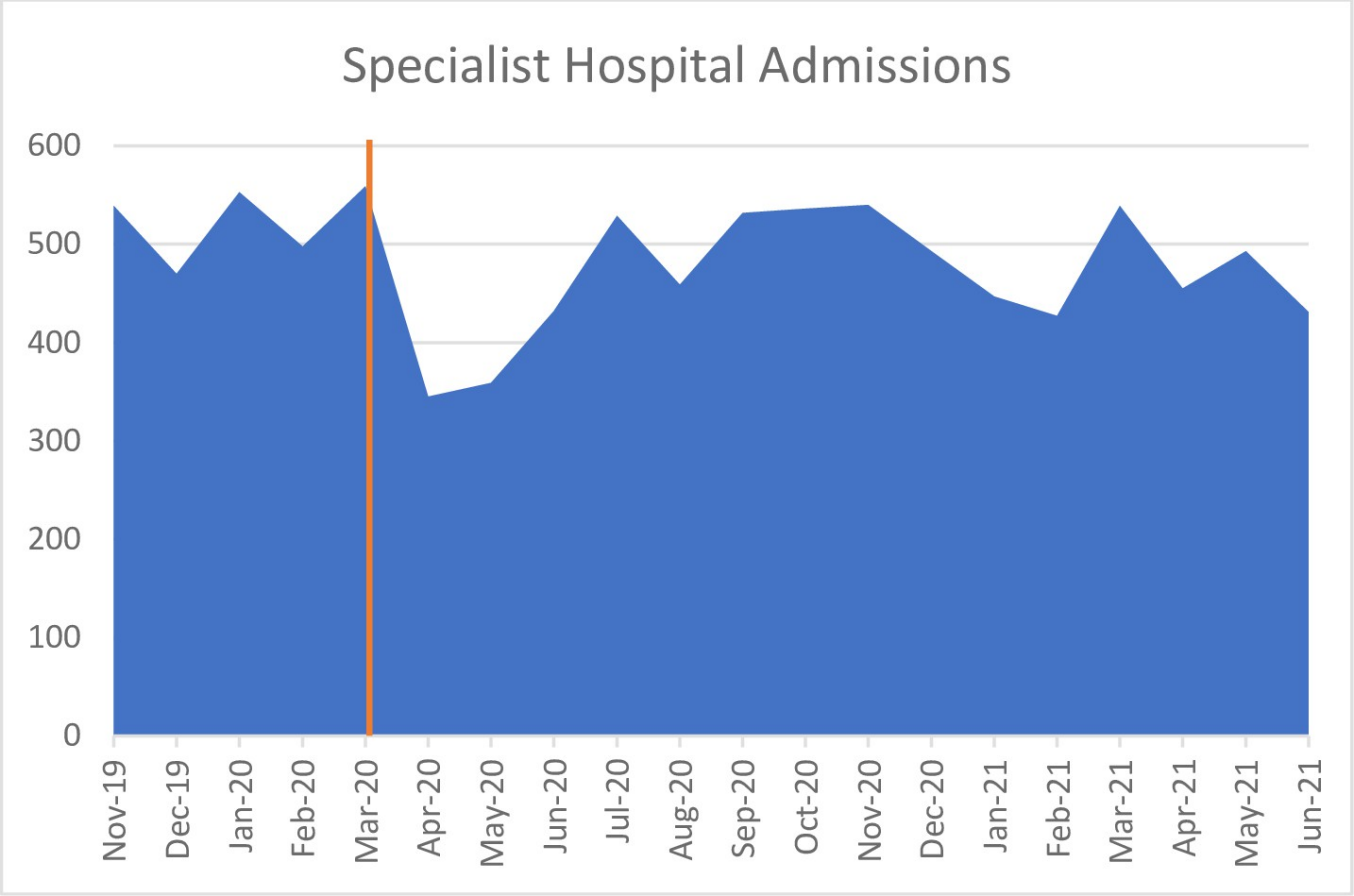
Specialist Hospital Admissions from November 2019 –June 2021 [26].

*Specialist psychiatric hospitals*. DHIS data demonstrated a marked decrease in admissions to specialist hospitals in the province when South Africa went into level 5 lockdown on 26 March 2020. Admissions dropped from 559 patients in March 2020 to 345 in April 2020 (Fig 1). Although no specialist hospitals closed, emergency acute admissions were prioritized, in-patients were discharged early, and all voluntary admissions were turned away.

*“We continued admitting but we just had a higher threshold for admission of patients*. *Just a small example*, *if somebody with autism and depression wanted to come in for ECT [electro-convulsive therapy] electively*, *we could not take that because of the risk” (Specialist Hospital psychologist 119)*.

There were also reports that substance use psychosis and substance-related emergency presentations dropped due to the alcohol ban and curfews.

From June 2020, national restrictions were eased somewhat, and by August, admissions had returned to pre-Covid-19 numbers. However, this occurred over a very short period of time, resulting in what service providers described as a “mental health storm”, with dramatic increases in patient load that “felt like a tsunami” (Specialist Hospital psychologist 108).

“What we saw between Covid waves would be a kind of ‘psychiatry wave’: an increased number of patients relapsing because they had not been able to access treatment” (Specialist Hospital psychologist 119).

Hospitals attempted to cope with this by adding mattresses on floors and accelerating discharges, but these strategies were limited by safety concerns (Specialist Hospital Psychiatrist 101). Following this, there was an addition of 40 psychiatric beds at one specialist hospital, and at another the substance detoxification unit was utilized for additional psychiatric admissions (117).

**Fig 2:**
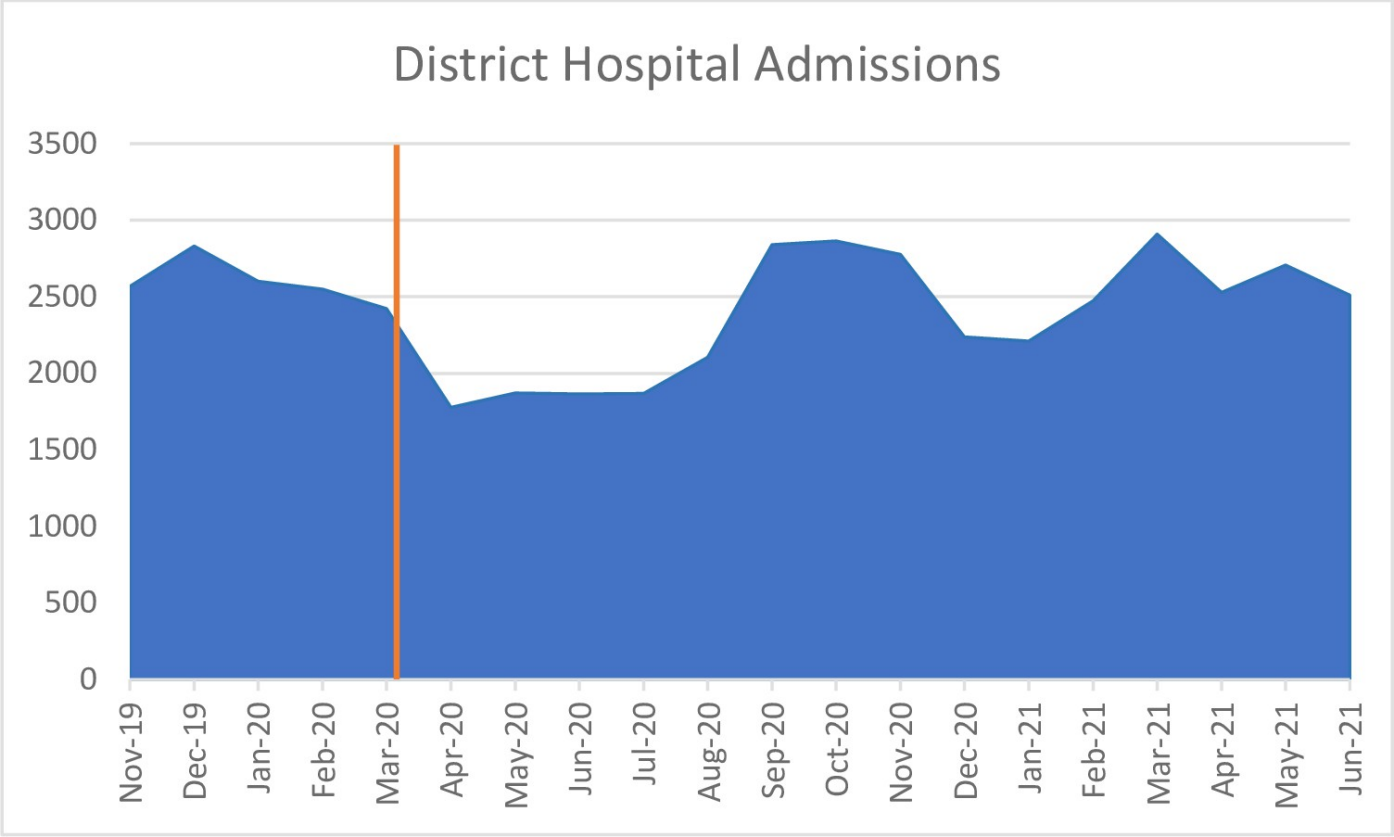
District hospital admissions from November 2019 –June 2021 [26].

*District hospitals*. There was also a clear decrease in admissions and clearance of psychiatric beds at district hospitals to make way for Covid-19 care. Admissions went from 2423 patients in the province in March 2020 to 1779 patients in April 2020 (Fig 2).

*“The plan was to empty the hospitals*, *to make beds for emergencies*. *So*, *we had to discharge people [who were] really sick and hope for the best” (District Psychiatrist 106)*.

In response to the increased need, in May 2020, additional psychiatric beds were added at district hospitals. Admissions then rose dramatically in August 2020 but dropped again during the second Covid-19 wave and lockdown in December-January 2021.

Participants reported that people with psychosis who presented at district hospitals would “not be able to receive containment because they closed the units” (Specialist Hospital psychologist 108). Patients were referred straight to specialist hospitals, but this increased the pressure for beds at hospitals that were already over capacity. A stakeholder related that patients from district hospitals were then ‘warehoused’ at specialist hospitals without appropriate treatment due to lack of specialists and closure of wards:

*“So*, *they basically get parked and they wait [which meant at one hospital*, *the] waiting list has gone from an average of about 25 to 89*. *Because the patients come and as soon as they come in they get shunted around with very little actual clear intervention to get them treated and move out*. *So our waiting list has just ballooned” (Specialist Hospital Psychiatrist 113)*.

This participant further highlighted that despite this increase in need, *“*our staff complement has remained the same as it has always been for the last ten years. Despite the massive increase in demand, we haven’t had an increase in resources either from an infrastructural perspective or from personnel” (Specialist Hospital Psychiatrist 113).

#### 2.2. Outpatient psychiatric medical visits

**Fig 3:**
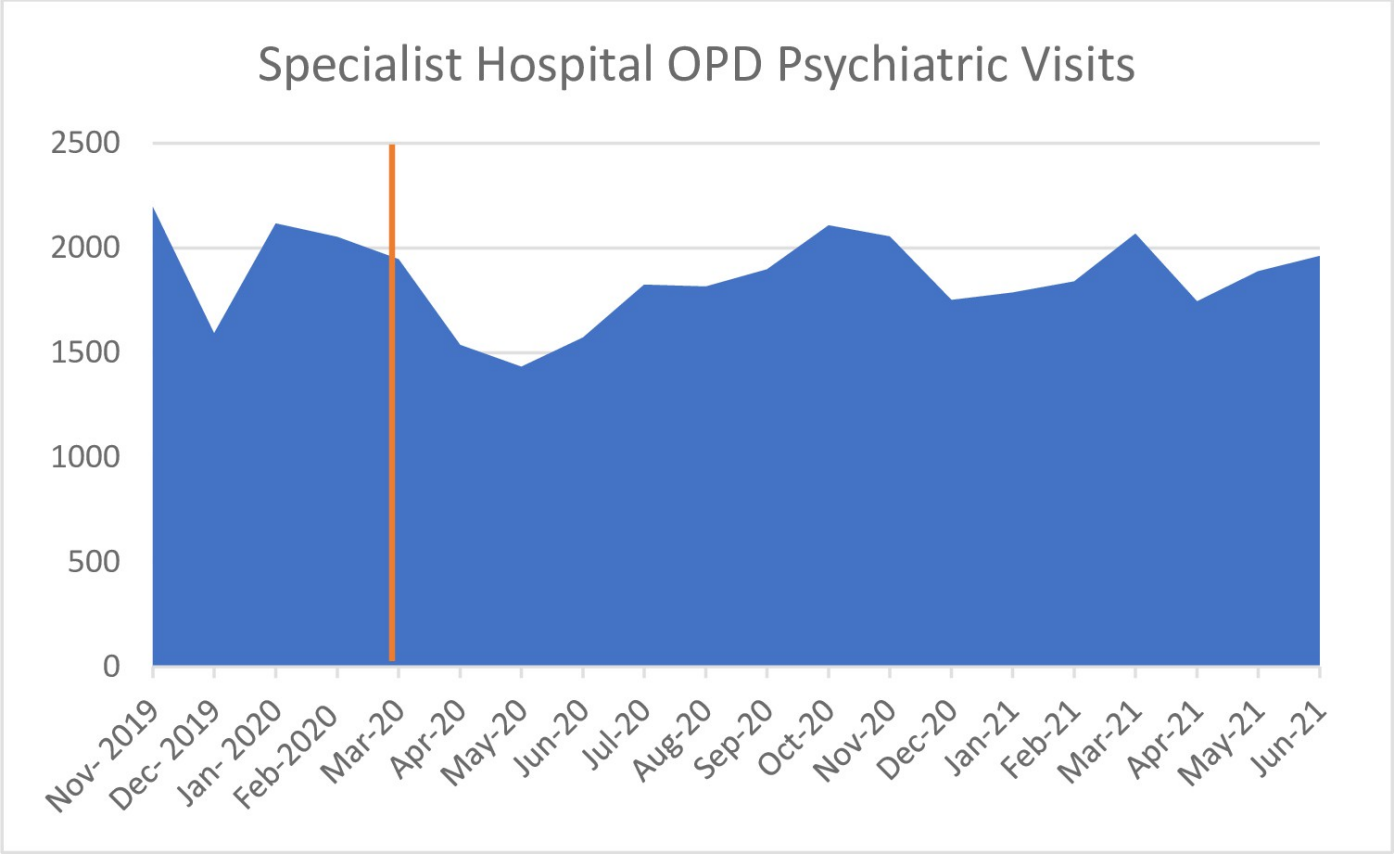
Specialist hospital OPD psychiatric visits from November 2019 –June 2021 [26].

*Specialist hospitals*. Outpatient (OPD) psychiatric medical visits at specialist hospitals decreased from 1946 visits in March 2020 to 1537 in April 2020 (Fig 3), with specialists also prioritizing emergencies. Appointments were also spaced out to longer time periods between visits for patients.

*“This was a numbers decision*, *to limit the numbers of patients in the waiting area and at the pharmacy* … *[In addition]*, *we could provide up to three months’ scripts at a time and the pharmacy was dispensing greater than a month’s worth of medication in order to accommodate that” (Specialist Hospital psychologist 119)*.

Unlike the psychiatric admissions, the easing of lockdown restrictions did not lead to a return to pre-Covid-19 OPD visits.

**Fig 4:**
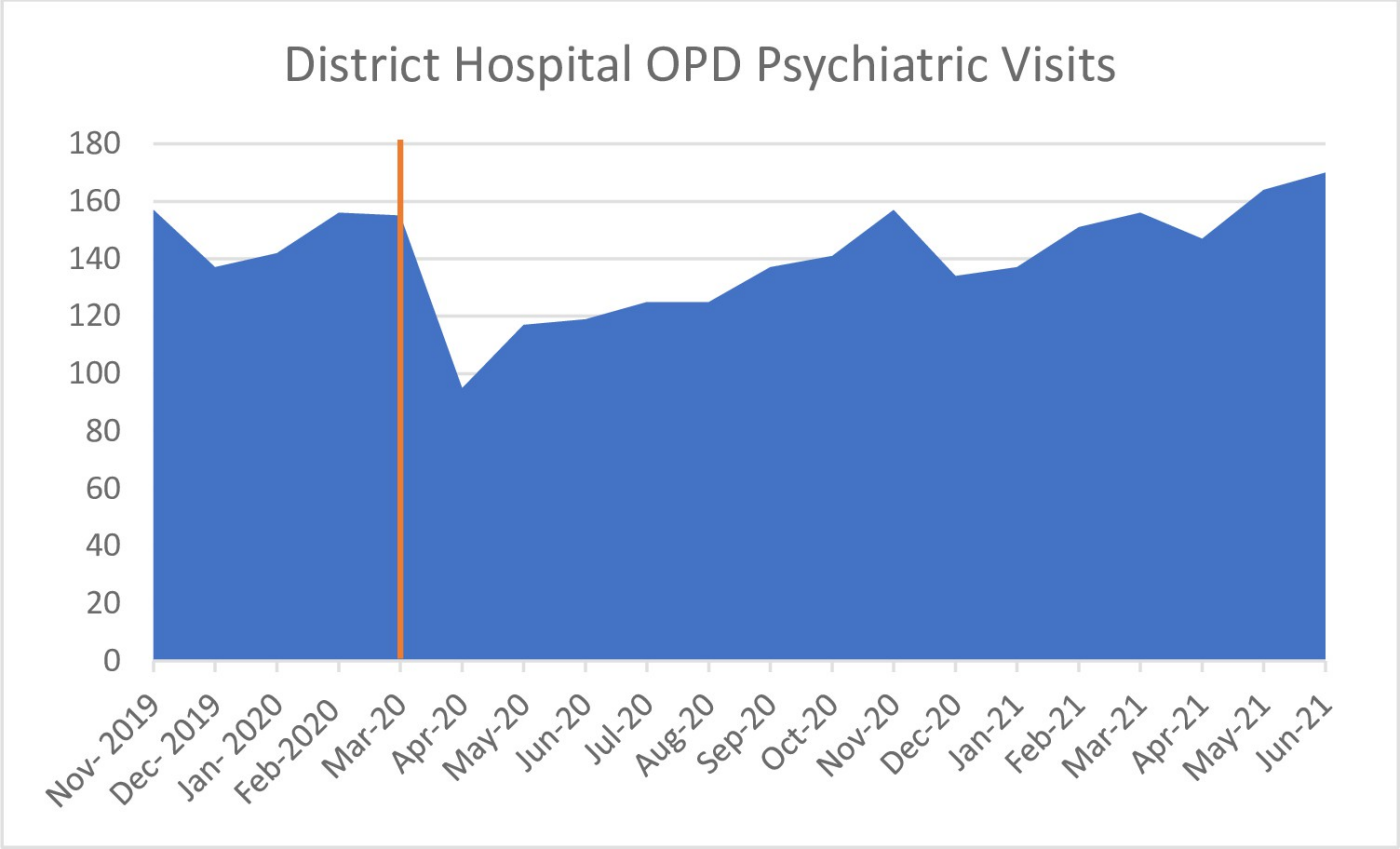
District Hospital OPD medical visits from November 2019 –June 2021 [26].

*District hospitals*. District Hospitals were given directives to reduce outpatient psychiatric medical visits to 30%, which affected visit numbers (Fig 4). Qualitative and narrative stakeholders confirmed that rooms that were usually used to deliver out-patient mental health care were used for Covid-19 and other acute care responses. Face-to-face contact with doctors, nurses and specialists was limited to emergency or acute cases. Patients were also given longer prescriptions and sent straight to pharmacies to collect their medication. One stakeholder illustrated the situation:

*“We were telling people not to come to hospital unless they absolutely had to*. *Even our outpatients were phoned and told not to come” (District Psychiatrist 106)*.

**Fig 5:**
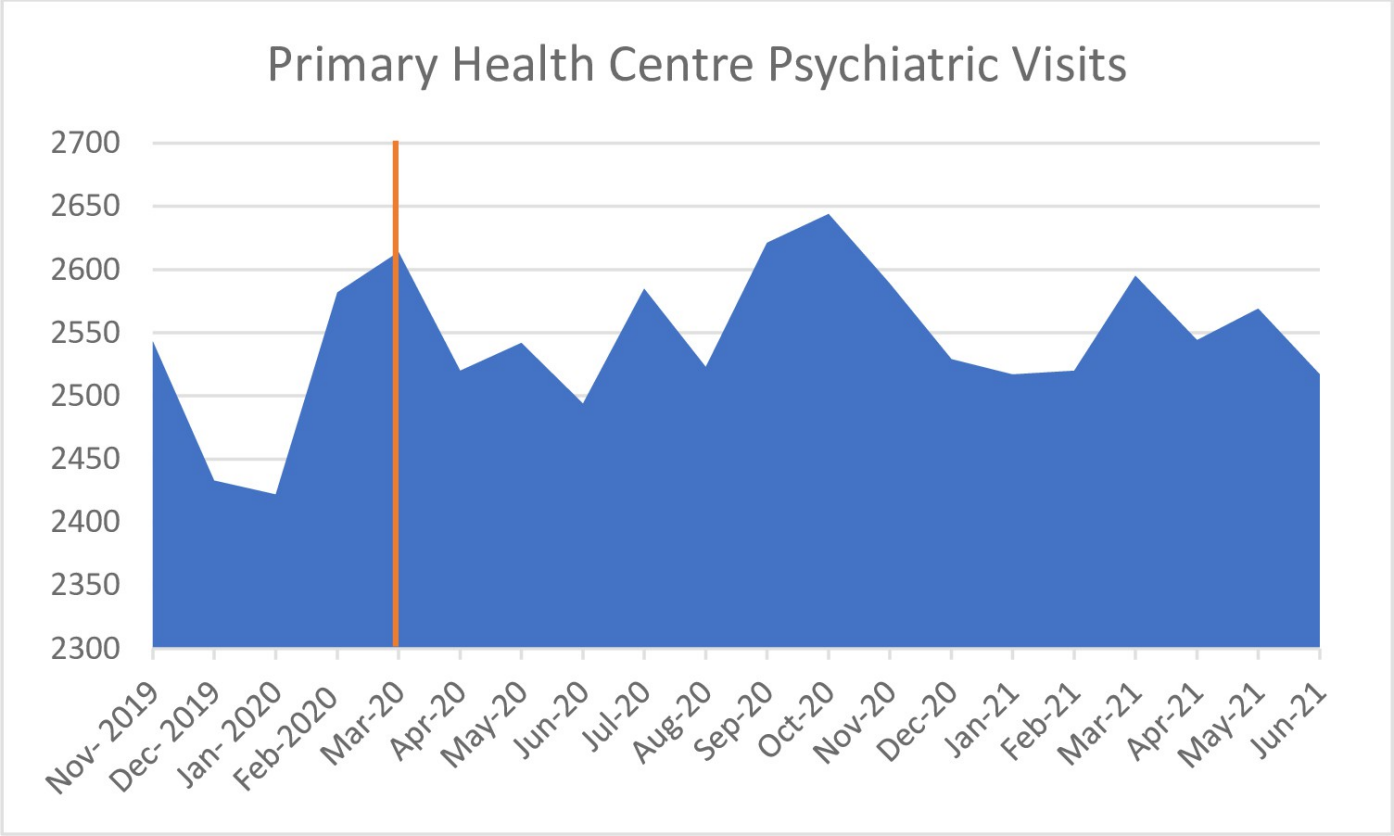
Primary Health Centre psychiatric visits from November 2019 –June 2021 [26].

*Primary Health Centres (PHC)*. DHIS outpatient psychiatric medical data for PHCs shows a slightly different trend to the hospital data, in that outpatient visits only reduced from 2614 in March to 2520 in April 2020 (Fig 5).

In the initial lockdown, doctors and community psychiatrists only saw patients face-to-face if they were emergency patients. All others were seen outside the PHC building by nurses and were sent straight to the pharmacies for medication dispensation (Specialist Hospital Psychiatrist 112). On a positive note, a province-wide programme was established to deliver chronic medication to patients’ homes, and chronic oral psychiatric medication was added to this medication list, enabling home delivery for some patients.

The regular community-based visits to chronic care patients by community health workers were also halted as they were “redeployed to do Covid screening in communities” (District Psychiatrist 106). Further, supervision of mental health nurses also stopped for a while because Covid-19 restrictions did not allow face-to-face supervision and they did not have mobile phone data to access zoom calls.

A notable factor that affected mental health service provision was the occurrence of extended periods of stock-outs of certain psychotropic medications. Prior to the pandemic, South Africa already faced stock-out challenges, particularly in rural areas. During the lockdown, the availability of medications such as lithium, haloperidol, and lorazepam was compromised “due to supply-chain issues” (Specialist Hospital Psychiatrist 101). This caused many adverse consequences and relapses for MHCUs, and issues for the psychiatrists and nurses who had to try and find replacement medications, which were not optimal for their patients and which required new scripts every time stockouts occurred.

### 3) Interruption in provision of therapeutic services

**Fig 6:**
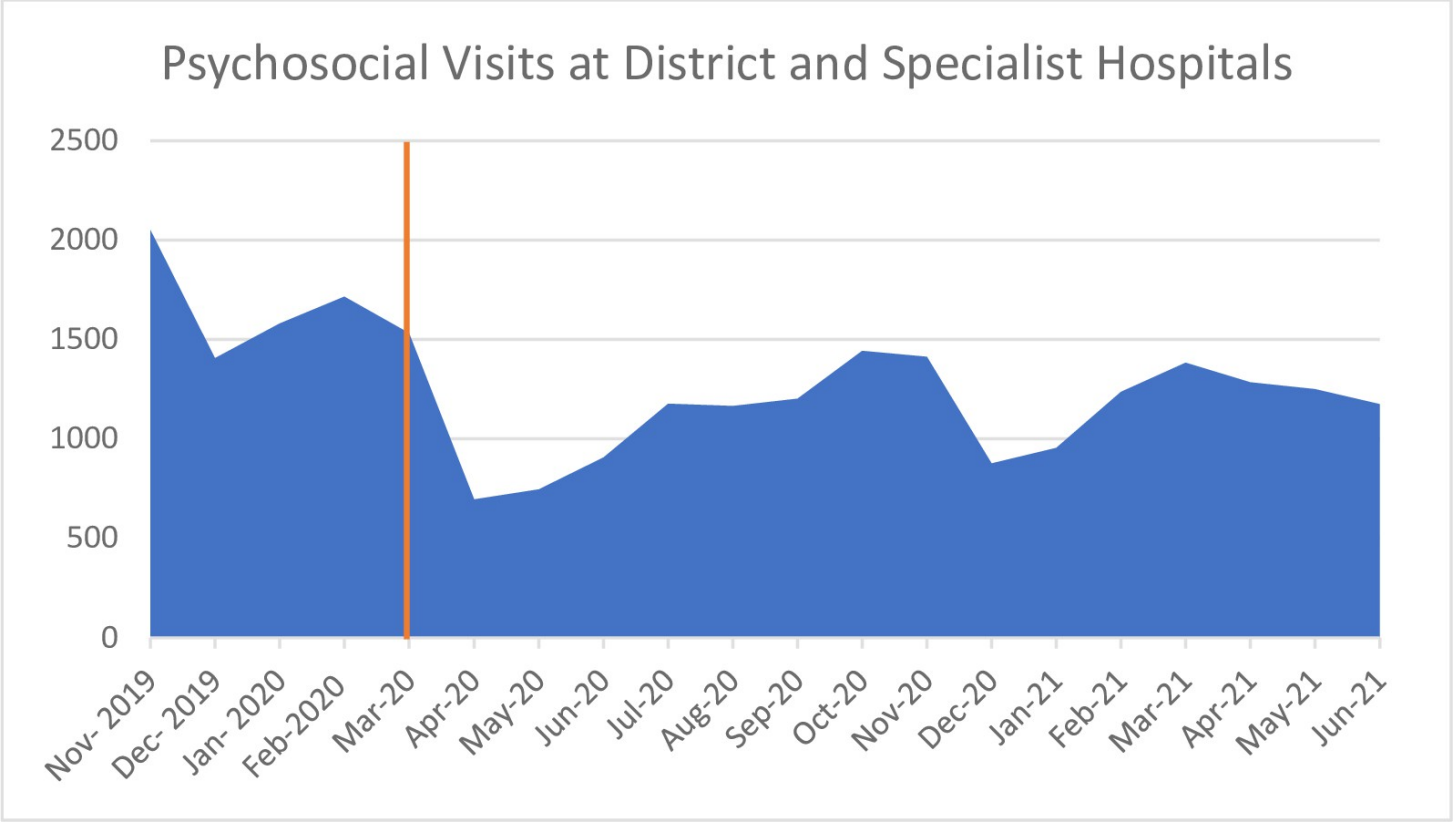
District and specialist psychosocial visits from November 2019 –June 2021 [26].

One of the more disheartening sets of findings from the various data sources was the closure of inpatient therapeutic wards and out-patient psychosocial rehabilitation and therapeutic programmes at all levels of care. Therapeutic visit numbers reduced from 1533 in March 2020 to 695 in April 2020 when lockdown started (Fig 6). By June 2021, at the time of writing, treatment numbers had still not returned to pre-Covid-19 levels.

Participants reported that psychological treatments and psychosocial group therapies were put on hold in all settings in order to prevent face-to-face contact, to “make bed space, and to redeploy staff to Covid care wards”. Hospitals were “compelled to close down the therapeutic units because there was no other way to get staffing for the Covid positive wards” (Specialist Hospital psychiatrist 113). Staff from therapeutic wards were deployed to Covid-19 wards to manage the influx of medical patients and keep positive and negative patients separate. This meant that specialists had to “reduce regular psychiatry work to manage Covid wards” (District Psychiatrist 120), and some registrars training in psychiatry were sent to internal medicine wards (Specialist Hospital psychiatrist 119).

The affected treatments included psychosocial programmes for people with psychotic disorders, common mental disorders, PTSD, personality disorders, anorexia, substance abuse problems, and for elderly patients. Interview participants confirmed in June 2021 that services had not returned to pre-Covid-19 levels, and that one of the reasons for this has been the successive Covid-19 waves and ensuing lockdown restrictions. Several stakeholders highlighted the negative implications of the prolonged closure of psychiatric wards and the ad-hoc planning that was required as a result of Covid-19. This is encapsulated by the following statement:

*“The therapeutic and neuro psychiatric ward was taken over by medicine*, *so there was no therapeutic programme*, *which was initially okay*, *but what happens to the anorexic patients who need care*? *So*, *they all became very sick*, *and the geriatric patients in the psychiatric ward wound up in the general acute wards*. *It just felt like they had not been thought about thoughtfully and mindfully when services were planned” (Specialist Hospital psychiatrist 109)*.

Deployment of staff placed increased pressure on those managing psychiatric patients, and on an already strained system. Stakeholders reflected on the situation as follows:

*“In the hospitals we are doing our best*, *but the infrastructure is terrible*, *we are understaffed*, *patients aren’t treated well*, *they aren’t treated with dignity*. *I think that just got exacerbated because as I said you withdrew services as it was*. *People got even less*. *It got more dire*. *I don’t think that anybody thought about the mentally ill… If you already have a service that has been grossly underfunded*, *if you are removing more resources*, *the knock-on effect of that is just profound… Things just got harder*, *and they were already hard” (District Psychiatrist 106)*.

*“What Covid has done is it has pulled the scab off something that which would never have been allowed*. *But it has exposed*. *It exposed the gross inequity or the under resourcing of the system*. *There is absolutely no slack in it*. *There is no give in it” (District Psychiatrist 114)*.

### 4) Minimal provision for MHCUs in legislation

Questions regarding national policy development revealed that Covid-19 policies were developed with minimal consideration for MHCUs and their vulnerabilities. Similarly, policies concerned with mitigating the social and economic impact of Covid-19 on households did not consider MHCUs. One specialist also related that “there were no policies in place for patients with substance use disorders. So there was no legislation protecting people who use drugs. The prohibitions really impacted those people in a negative way” (Specialist Hospital psychiatrist 109).

The National DOH published guidelines on mental health interventions during Covid-19 [27], and a protocol was created by the Western Cape DOH for referral processes and roles of each specialist and district hospital for managing Covid-19 positive cases. In these policies and guidelines, each mental health facility was responsible for managing their own Covid-19 protocols. Hospitals had to make provision for quarantine spaces for MHCUs in each catchment area (public sector servant 117), but these had limited oxygen supplies and bed space. The lack of adequate psychiatric quarantine spaces meant that at various time points suspected Covid-19 positive psychiatric patients had to be admitted into psychiatric wards. This resulted in a number of outbreaks of Covid-19 in psychiatric wards. Due to these occurrences, referring NGOs were advised to keep patients in their homes and cared for by family members unless the individual was too acutely unwell to be managed in the home setting (NGO service provider 104).

Psychiatric hospitals and group homes also were not able to enforce social distancing, mask-wearing, and appropriate Covid-19 hygiene standards due to the mobile nature of psychiatric patients and the sharing of wards, dining rooms, living spaces, and bathrooms.

### 5) Increased mental health needs of health care workers

The data also highlighted the drastic rise in mental health care needs of HCWs across all levels of care. Many were reported to be suffering from burnout as a result of the pressures from Covid-19. These needs have “increased tremendously”, and there seemed to be “no reprieve for people working in mental health” (119).

*“They have been exposed to the suffering and deaths of Covid patients*, *and also terrified of the fact that they could possibly contract the virus*, *but yet had to continue working and not be able to see their families due to being isolated*. *They lost colleagues and loved ones*. *Furthermore*, *they provided care and comfort to dying patients*, *in the absence of patients’ loved ones*. *They worked long hours and continuously put their own lives at risk*. *Compassion fatigue and burnout is a real threat to the mental health of frontline workers*. *Grief*, *loss and death became a trauma we were more exposed to” (NGO service provider 104)*.

In response to this need informal support networks were created within facilities, with reports of peer-support interactions and group ‘check-ins’ at many different hospitals. A ‘Healthcare Workers Care Network Initiative’ was set up but not widely adopted. Many stakeholders related that providing psychological support to frontline workers was vital for the wellbeing of facility staff.

### 6) Innovations to the provision of care

With the many challenges that Covid-19 and the lockdowns created for mental health service provision, facilities and NGOs had to create innovations to adapt to the changing environment. Participants provided many examples of innovations that were implemented. These are documented in the hope that they may be useful for further Covid-19 outbreaks or in situations of future health emergencies or pandemics.

Participants also explained that oral psychotropic medication had been added to the essential chronic medication list. This was a major achievement that enabled the delivery of these medications to patients’ homes [28], and will greatly reduce travel costs and time for these patients going forward. For those who did have to come to clinical centres, prescriptions of medications for chronic psychiatric patients were provided for up to three months, so that patients did not have to come to the hospital or clinic every month.

Those who used to see care users face-to-face implemented hybrid telephone and face-to-face consultations with care users and their families. These worked particularly well for chronic patients. This prolonged the time period between consultations, reducing patient load but also assisting patients who cannot afford to visit the clinic/hospital/NGO every month. One facility also introduced virtual patient-family liaisons through the use of ‘Quinton the Robot’. This was an iPad on an arm on wheels, where patient contact with family members was facilitated without a nurse having to be in contact with the patient. This has long term possibilities for other applications beyond pandemics, such as virtual consulting for high-risk patients such as bone marrow transplants and open burns.

Teaching and learning opportunities for interns, registrars, and NGO staff were also created virtually. This included conducting ward rounds virtually. This enabled supervisors to reach more interns across districts and reduce the load at certain hospitals. This format is also useful going forward given the high burden that supervisors and specialists carry in public hospitals, and in addition reducing travel time for interns and students. Guidelines for conducting virtual or telephonic psychotherapy were also created for psychiatry and psychology registrars and NGO social workers. Last, NGOs responded to the need for information and support for care users through contact with them via WhatsApp, phone calls, or SMS’s, making use of voice notes, infographics, and easy-to-read documents on information on Covid-19 and on mental health. These networks are essential for the transfer of information and support to the public and may be relied upon when needed again in the future.

## Discussion

Several key findings emerged from this study. First, the consequences of the various lockdown restrictions resulted in an increased need for mental health services and information, and in many known MHCUs experiencing relapse. This was evidenced by the substantial increase in calls to the SADAG national mental health hotlines, and the ‘mental health storm’ experienced by providers. Consistent with the available literature [29] the strict measures to prevent the spread of Covid-19 in South Africa and in other LMICs such as school closures, re-allocation of health resources, and restrained economic opportunities, exacerbated experiences of distress and mental illness. Given these are known determinants of mental illness this may have long term impacts on the mental health of South Africans [30].

Further, the measures taken to mitigate the spread of Covid-19 led to a loss of financial, food, and health security for already vulnerable MHCUs, and compounded anxiety around Covid-19. Due to strict travel restrictions, lack of money for transport, and fear of catching Covid-19, many MHCUs were unable or afraid to go to clinics and hospitals to obtain their medication. Our study also found that the five-month cigarette ban compounded psychological distress and led to relapses of more severe substance abuse for many MHCUs. This occurred while detoxification and substance abuse centres were closed, a phenomenon that was also reported by 30% of countries in a World Health Organization (WHO) survey of 130 Member States [31]. In India, where there was also a prohibition of alcohol sales, there were also reported influxes of patients facing substance use withdrawal resulting in admission [32]. This put further pressure on the system when services returned to normal.

Second, the availability of mental health services at all levels of care decreased substantially during the lockdowns. Medication provision was prioritised, patients were sent straight to pharmacies without consultations, and some in-patients were discharged prematurely. In order to limit contact, the provision of longer prescriptions for chronic patients was employed, a strategy also implemented in other countries [33]. Psychiatric staff not required in emergency response were re-directed to Covid-related care and several psychiatry wards were closed to accommodate Covid-19 patients. The mental health sector was already under severe strain, with insufficient human, financial, and infrastructural resources prior to Covid-19 [34]. Participants in this study related that lockdown measures and reallocation of funds intensified this situation, to the degree that instances of human rights abuses were even reported due to lacking resources.

The disruption in the availability of mental health services as a result of Covid-19 was reported by other countries in Africa, despite WHO recommendations that mental health care should be included within ‘essential services’, with 75% of MNS-related services being completely or partially disrupted in 57% of 28 African countries [31]. Globally, high-income countries reported lower levels of disruption of services, with only one quarter (24%) affected. The inequities within the South African mental health system were therefore exacerbated, heightening the already massive treatment gap in South Africa [35, 36]. DHIS data showed fewer disruptions to visits at PHCs, which is surprising given the limited integration of mental health services into primary care in South Africa. This may have been due to nurses registering medication collection outside of PHC buildings as service visits.

During lockdowns, all in-person psychosocial and therapeutic services were halted, as they were not prioritised as an ‘essential service’. This persisted beyond reasonable expectations for service closures, and in June 2021, therapeutic service provision had still not returned to pre-Covid-19 levels. This had and has significant implications for out-patient MHCUs, and demonstrates a systemic undervaluing of non-biomedical care, warranting further investigation.

In efforts to mitigate the impacts of this, service providers and NGOs attempted to contact patients via telephone or WhatsApp but this was limited by patients’ access to telephones, mobile data and airtime. Restricted phone, electricity, and WiFi access similarly restricts MHCU access to digital services in many other LMICs [30, 37]. NGOs also responded by designing and implementing training for service providers to conduct online and telephonic consultations (for example, Cape Mental Health, and the South African Federation for Mental Health).

There are examples of various countries shifting to remote services or hybrid approaches, such as creating suicide and mental health helplines, phone-based services, and video services over smartphones [30]. However, these were largely in upper- middle-income countries, and were not implemented at scale. Some established interventions are being adapted for remote delivery in LMICs, such as the Problem Management Plus intervention for humanitarian settings [38] and the Friendship Bench intervention in Zimbabwe [39]. In addition, several evidence-based mental health interventions in South Africa may be delivered remotely [40–42].

Third, stakeholders reported a decrease in the quality of services that remained available, and this included stockouts of psychiatric medication. Further, the national Covid-19 policies did not make special provisions for MHCUs or for managing Covid-19 in psychiatric institutions, resulting in increased neglect of an already vulnerable population. This could be viewed as a form of structural stigma towards those with mental health conditions. Under pressure to treat psychiatric emergency patients, suspected positive patients were on occasion placed in non-Covid-19 psychiatric wards, leading to instances of human rights violations. In addition, there were challenges in enforcing infection control measures amongst inpatients within psychiatric hospitals, an issue also faced in other countries [43, 44].

Fourth, consistent with studies conducted globally [45–47], a critical gap in the current health service is mental health support for frontline workers; a gap exposed and exacerbated by Covid-19 [30]. Prior to Covid-19 the needs of HCWs were neglected, but these needs were exacerbated by having to care for co-workers, isolation from family members, being quarantined, and inadequate access to personal protective equipment [48, 49]. A systematic review examining mental distress and disorders amongst HCWs during the pandemic across 19 countries found a pooled prevalence of PTSD to be 49%, anxiety 40%, depression 37%, and distress to be 37% [50]. It is vital that psychosocial support personnel are employed *in* health facilities to assist staff and prevent further burnout and trauma [29]. Hoare and Frenkel [51] recommend ‘collegial interventions’ at hospitals, which involves including mental health professionals in Covid-19 frontline teams, to facilitate trust and shared experiences of trauma and mental health needs.

The findings from this study led to a number of recommendations for the National and Provincial Departments of Health with regard to policy formation and resource provision for future pandemics and health emergencies. Many of the recommendations highlighted by stakeholders, such as improving funding, staff, and resources, are consistent with those set out in the mental health policy and strategic plan [52], again highlighting that the mental health sector is neglected and that the plan was not adopted. However, there were also recommendations put forward that were more specific to responding to a pandemic. These included: involving MHCUs in the development of pandemic responses; adding delivery of oral psychotropic medication and longer prescriptions of medication into routine care for MHCUs; the establishment of an intersectoral mental health emergency response plan across specialist and district hospitals, PHC and community mental health services, to co-ordinate bio-psychosocial mental health services, resources, funding, and personnel; creation of policies for managing Covid-19 and other pandemics in psychiatric facilities; the establishment of field hospitals for infected MHCUs so that therapeutic wards remain open and are not used as spaces for general pandemic care; train and resource mental health care workers to contact and consult patients telephonically; create opportunities for the continuation of a ‘hybrid’ model for chronic care patients who may not need to see specialists every month; and last, to re-classify psychological treatments as an essential service that cannot be shut down [16].

The findings of this study should be considered in light of a number of limitations. First, we were not able to access MHCUs for their perspectives due to lockdown restrictions. Second, there are concerns regarding the variability in DHIS data quality among the facilities. Third, there was a relatively small sample size, which was affected somewhat by service providers already being overburdened with demands on their time. Last, due to limited funding resources, this study was limited to the Western Cape Province. Sampling of service providers in other provinces is likely to have painted a different picture, as the Western Cape has the smallest treatment gap given the number of mental healthcare providers per population than the other provinces [9].

## Conclusion

This study provides narrative evidence that mental health service provision in the Western Cape was significantly and negatively impacted by Covid-19 and the related lockdowns, particularly during the initial five-week ‘hard lockdown’. This had numerous ramifications for MHCUs who were not able to access their normal services. Combined with an loss of financial, food, and health security for already vulnerable MHCUs, this led to a dramatic rise in relapses of mental illnesses. The lockdown regulations drastically reduced clinical service provision and psychosocial therapy at health facilities and re-directed psychiatric staff to Covid-19 care. Medication provision was prioritised, patients were sent straight to pharmacies, and some in-patients were discharged prematurely, thus worsening the already neglected mental health sector, and exacerbating the treatment gap for mental illnesses in the Western Cape. The mental health needs of frontline workers concurrently increased, with little-to-no support available to them. Following the initial lockdown, various modifications were made to mental health services, and these have already demonstrated improvements in access to medication, clinical, and therapeutic services, through the use of virtual platforms. The recommendations provided by participants have the potential to guide policy and strategic plans for mental health care service provision for South Africa and other LMICs in the event of future public health emergencies. These include classifying psychological treatments as essential services, establishing an intersectoral mental health emergency response plan, involving mental health care users in the development of pandemic responses, and creating policies for managing health emergencies in psychiatric facilities. This study further highlights the urgent need to prioritize mental illnesses and increase the provision of funding and resources for the mental health sector.
